# Fibroblasts in Diabetic Foot Ulcers

**DOI:** 10.3390/ijms25042172

**Published:** 2024-02-11

**Authors:** Francesca A. Voza, Carlos Theodore Huerta, Nga Le, Hongwei Shao, Antoine Ribieras, Yulexi Ortiz, Carl Atkinson, Tiago Machuca, Zhao-Jun Liu, Omaida C. Velazquez

**Affiliations:** 1 DeWitt Daughtry Family Department of Surgery, University of Miami Miller School of Medicine, Miami, FL 33136, USA; fav38@med.miami.edu (F.A.V.); cth62@med.miami.edu (C.T.H.); hshao2@med.miami.edu (H.S.); antoine.ribieras@med.miami.edu (A.R.); yyo2@med.miami.edu (Y.O.); t.machuca@med.miami.edu (T.M.); 2 Vascular Biology Institute, University of Miami Miller School of Medicine, Miami, FL 33136, USA; 3 Department of Biochemistry & Molecular Biology, University of Miami Miller School of Medicine, Miami, FL 33136, USA; 4 Department of Internal Medicine, Division of Pulmonary Critical Care & Sleep Medicine, University of Florida, Gainesville, FL 32611, USA; carl.atkinson@medicine.ufl.edu

**Keywords:** fibroblast, myofibroblast, diabetic foot ulcer, diabetic wound, chronic wound, chronic inflammation

## Abstract

Fibroblasts are stromal cells ubiquitously distributed in the body of nearly every organ tissue. These cells were previously considered to be “passive cells”, solely responsible for ensuring the turnover of the extracellular matrix (ECM). However, their versatility, including their ability to switch phenotypes in response to tissue injury and dynamic activity in the maintenance of tissue specific homeostasis and integrity have been recently revealed by the innovation of technological tools such as genetically modified mouse models and single cell analysis. These highly plastic and heterogeneous cells equipped with multifaceted functions including the regulation of angiogenesis, inflammation as well as their innate stemness characteristics, play a central role in the delicately regulated process of wound healing. Fibroblast dysregulation underlies many chronic conditions, including cardiovascular diseases, cancer, inflammatory diseases, and diabetes mellitus (DM), which represent the current major causes of morbidity and mortality worldwide. Diabetic foot ulcer (DFU), one of the most severe complications of DM affects 40 to 60 million people. Chronic non-healing DFU wounds expose patients to substantial sequelae including infections, gangrene, amputation, and death. A complete understanding of the pathophysiology of DFU and targeting pathways involved in the dysregulation of fibroblasts are required for the development of innovative new therapeutic treatments, critically needed for these patients.

## 1. Introduction

Fibroblasts are ubiquitously distributed in the human body, located in the interstitial space of most organs. These cells have received increased attention as recent studies have revealed the pivotal role they play in a wide range of disease pathophysiology ranging from malignancy, heart failure, fibrotic kidney to liver diseases, as well as in the tightly regulated process of wound healing. The treatment of chronic wounds with impaired healing potential is still subject to high failure rates. Treatment of diabetic foot ulcer (DFU) in particular, the most common comorbid condition of Type 2 Diabetes Mellitus (T2DM), remains very challenging. These non-healing wounds develop in up to 25% of T2DM patients, which represents 40 to 60 million people, and significantly reduce patients’ quality of life [1]. DFU patients are subject to high rates of impaired mobility, necrosis, infections, gangrene, and even death from these non-healing wounds [2]. Many factors are responsible for the pathophysiology of DFU including infections, reactive oxygen species (ROS), poor perfusion and advanced glycation end products (AGEs). Current medical treatment regimens include debridement of devitalized tissues, local wound care dressings, off-loading, glucose control, negative pressure wound therapy, skin grafting, bioengineered skin, and energy therapy [2,3]. A significant number of patients have exhausted all these options and are not suitable for surgical revascularization interventions. These “failure to standard treatments” patients face substantial risks of limb amputation, which is associated with a survival rate of 50% at five years [4]. In addition to this disease burden, the annual cost of these treatments for DFU to the American healthcare system are estimated to rise between 9 to 13 billion dollars [5]. As a result, innovative treatments are critically needed to palliate this serious public health issue. The purpose of this review is to provide an understanding of the functioning of fibroblasts and the mechanisms involved in the pathophysiology of DFU, which can aid in the identification of potential targets for novel therapeutics.

## 2. Fibroblasts: A Heterogenous Cell Population with High Cellular Plasticity and Broad Functionality

Fibroblasts are mesenchymal stem cell-like cells, present in the stroma of most organ tissues of the body. These cells were previously considered “passive” solely maintaining the architecture of the tissue-specific extracellular matrix (ECM), which provides a stable micro-environment essential to the appropriate functioning of physiologic activities within the organ [6]. However, recent studies have highlighted the dynamic and central role of fibroblasts in maintaining tissue specific homeostasis and integrity [7,8]. New insights have shed more light on the high plasticity [7,8] and heterogeneity of the fibroblasts [7,9,10]. Over the past two decades, more advanced techniques such as single cell analysis have enabled us to identify through transcriptomic and epigenomic profiles, different fibroblast lineages [11,12,13,14,15] with different phenotypes and a broad range of functions [13,16,17]. These cells not only exhibit a diverse range of phenotypes but also possess the ability to switch their phenotypic characteristics through processes like trans-differentiation, leading to the development of cell types such as myofibroblasts and adipocytes [12,18]. Dermal fibroblasts originating from the largest and most accessible organ exposed to daily insults from the external environment, have been the most studied [18,19,20], primarily utilizing the mouse model.

### 2.1. Tissue Specificity and Heterogeneity

Fibroblasts can be classified into different lineages based on their tissue of origin and specialized functions [7,21]. They also retain a degree of capacity for dedifferentiation, a cellular process in which cells lose their specialized characteristics and transition from a partially or terminally differentiated state to a less differentiated stage within their own lineage. Fibroblasts continue to mature in the mesenchyme where they are in a quiescent state in normal physiologic conditions, dynamically involved in the maintenance of the ECM, as well as structural homeostasis and integrity of a tissue. They display significant heterogeneity from one tissue to another but also within the same tissue, in terms of morphology, biomarkers, gene expression profiles, epigenetic modifications, and signaling pathways. This heterogeneity arises due to various factors, including their tissue-specific functions, interactions with neighboring cells, and exposure to different microenvironment cues [7,10,21].

Many of the cell surface markers used to identify fibroblasts subtypes are not specific to the fibroblast population and the heterogeneity within and between organs represents a challenge to the identification of fibroblast populations with the use of current markers utilized [8,10,13,22]. Advances in single-cell sequencing technologies have revolutionized our ability to study cellular heterogeneity [13,16] and identify distinct subtypes of fibroblasts with the characterization of additional markers, with higher resolution [11,12]. Whereas traditional bulk RNA-seq analysis provides an average gene expression profile of a cell population [7,8,12,14], single cell RNA-sequencing (scRNA-seq) allows for the capture and analysis of gene expression profiles from individual fibroblast cells [11,12,13,14,15,17]. In addition to transcriptional signatures, scRNA-seq provides valuable insights into molecular characteristics such as additional markers that define fibroblast subtypes and their associated pathways [13,16]. These markers can include membrane bound molecules, signaling molecules, extracellular matrix components, and transcription factors. This approach has enabled the discovery of fibroblast subtypes with unique functional properties and roles in tissue development, repair, and disease. The different functional properties of fibroblasts were first investigated within the context of tumor microenvironments where Cancer-Associated Fibroblasts (CAFs) subtypes were characterized in breast and pancreatic tumor models [23,24]. CAFs are fibroblast-like cells that are recruited to the tumor microenvironment and play a crucial role in tumor progression. Four different subtypes denoted as CAF-S1 to CAF-S4 for example, were identified in human breast adenocarcinoma tumor samples [24,25] based on the expression of six markers, including integrin β1 (ITGB1 or CD29), αFAP, platelet-derived growth factor Receptor (PDGFR)-β, FSP-1, α-SMA, and caveolin 1 (Cav1) [24,25]. The utilization of high resolution scRNA-seq studies has led to better characterization of these distinct subpopulations of CAFs within tumors [24,26,27], based on differential expression of genes related to extracellular matrix remodeling, inflammation, and immune modulation. CAF-S1 and CAF-S4 for example were found to secrete TGF-β and CXCL12, activating Notch signaling in tumor cells, thereby promoting proliferation and invasion [27]. The CAF-S1 sub-type highlights the extensive range of functions exhibited by CAFs with the identification of eight new clusters, each characterized by the expression of specific genes associated with distinct cellular processes. These clusters encompass various gene sets, such as those responsible for encoding ECM proteins (cluster 0, ecm-myCAFs), genes associated with the TGFβ signaling pathway (cluster 3, TGFβ-myCAFs), and genes involved in wound healing (cluster 4, wound-myCAFs), to name a few examples [24,25,27]. These markers can include membrane bound molecules, signaling molecules, extracellular matrix components, and transcription factors. Several functions within specific CAF subpopulations seem to be conserved across species, including extracellular matrix proteins synthesis, angiogenesis promotion, and immunomodulation [8,25]. Key signaling pathways like TGFβ- for instance, were found to be involved in the activation of CAFs in both human and mouse tumors. Nevertheless, significant differences exist in terms of heterogeneity, markers, and function properties. More diversity, for instance, has been described in human CAF subsets compared to mouse models, such as the CAF-1 subpopulation promoting immunosuppression that appears to be unique to humans, not reported in the mouse CAF population.

**Figure 1:**
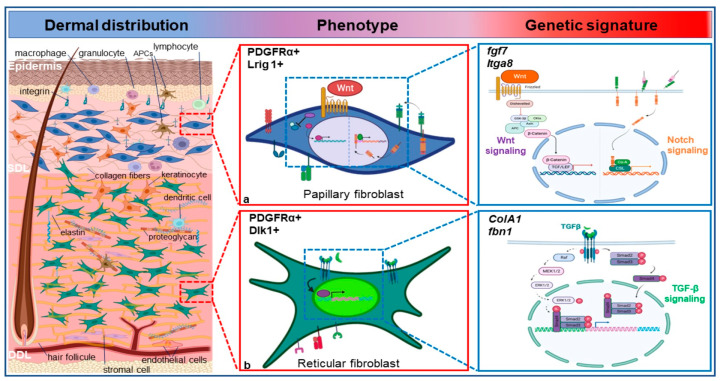
Heterogeneity of the dermal fibroblast population. Mouse dermal fibroblasts exhibit variations on the cellular and molecular levels based on their distribution in the dermis. The two main subpopulations are (**a**) papillary fibroblasts in the most superficial layer of the dermis and (**b**) reticular fibroblasts in the deeper layer of the dermis. These fibroblasts respond to different signaling pathways (simplified in figure) such as Wnt and Notch pathways in the papillary fibroblasts vs. TGF-β pathway in the reticular fibroblasts. They are characterized by differences in their phenotype with distinct morphology, surface markers expression, genetic signature, and epigenetic modifications. Similar markers were also characterized in the human papillary and reticular fibroblast populations. APC: Adenomatous Polyposis Coli; APCs: Antigen Presenting Cells; CSL: C-promoter binding factor 1 (CBF1), suppressor of hairless (Su(H)), lin-12 and glp1 (Lag1); Co-A: Co-enzyme -A; Col1A1: Collagen type I alpha 1; DLK1: Delta-like non-canonical Notch ligand 1; Fbn1: Fibrillin 1; Fgf-7: Fibroblast Growth Factor -7; FSP1: Fibroblast-specific protein 1; GSK: Glycogen synthase kinase; Itga8: Alpha-8 integrin; LRIG1: Leucine-rich repeats and immunoglobulin-like domains 1; MEK: Mitogen-activated extracellular signal-regulated kinase; NOS: Nitric oxide synthase; TCF/LEF: T-cell factor/lymphoid enhancer factor; TGF-β: Transforming Growth Factor-β; Wnt: Wingless-related integration site.

In a similar manner, further investigation into dermal fibroblasts’ heterogeneity based on scRNA-seq datasets has led to the characterization of two different subtypes of dermal fibroblasts, which are then divided into papillary dermal fibroblasts and reticular dermal fibroblasts subtypes in both mouse and human species [28,29] (Figure 1). Papillary and reticular fibroblasts show distinct morphology, functions, and gene expression profiles [29]. Papillary fibroblasts typically exhibit a more elongated and spindle-shaped appearance and are in the superficial layer of the dermis, known as the papillary dermis. These fibroblasts are primarily involved in tissue remodeling and wound healing processes. They contribute to the synthesis and deposition of extracellular matrix components, such as collagen and elastin fibers, promoting tissue integrity and elasticity. Additionally, papillary fibroblasts play a crucial role in regulating the local immune response through the secretion of cytokines and growth factors [30]. They have a more stellate or branched morphology compared to papillary fibroblasts. Reticular fibroblasts are primarily involved in synthesizing and maintaining the structural components of the dermal extracellular matrix [8,30]. Differences identified by Driskell et al. [28] in papillary and reticular murine dermal fibroblasts gene expression profiles, reflect their specialized roles and location within the dermis [28,29]. Murine papillary dermal fibroblasts studied were shown to express higher levels of genes involved in the crosstalk between the epidermis and the papillary dermis and contribute to the maintenance of epidermal integrity, such as keratinocyte growth factor or fibroblast growth factor (fgf)*-7* [30] and cell adhesion molecules like alpha-8 integrin (Itga8) [8]. Reticular dermal fibroblasts alternatively showed higher expression of genes associated with extracellular matrix production and organization. These genes include collagen type I alpha 1 (Col1A1) [29], fibrillin (Fbn1) [31], podoplanin (Pdpn) and delta-like non-canonical Notch ligand 1 (Dlk1) [8]. Similar pattern in human dermal fibroblasts were identified subsequently [32]. Yet, additional studies are required in both human and mouse species to define markers allowing a clear identification and purification of fibroblasts from each dermal layer [33]. Characterization of different signaling pathways selectively activated in each fibroblast subtype could contribute to the identification of fibroblasts subpopulations in addition to the expression of markers and receptors they are linked to. The Wnt signaling pathway, central to embryonic organogenesis, has been the most studied [8] and is involved in regulating multiple cellular processes, including dermal fibroblast functions in wound healing [9,34,35,36]. Studies on murine fibroblast subpopulations by Lichtenberg et al. [35] have demonstrated that papillary dermal fibroblasts will proliferate and generate ECM components once activated by the Wnt/β-catenin pathway, whereas reticular dermal fibroblasts will strongly respond to the TGF-β signaling and not to Wnt/β-catenin signaling. The activation of Wnt/β-catenin signaling in papillary fibroblasts is involved in their interaction with adjacent epidermal cells and contributes to the maintenance of epidermal integrity. It was also found that dermal papillary fibroblasts exhibit higher expression and activity of Notch receptors and downstream targets as well as higher levels of Hedgehog ligands and receptors compared to reticular fibroblasts. The Notch signaling pathway is known to play a critical role in cell fate determination and differentiation during development and tissue repair processes [37]. This pathway in papillary fibroblasts contributes to their interaction with neighboring cells, including keratinocytes [38], while Hedgehog signaling pathway suggests involvement in tissue organization and development [35].

Data from scRNA-seq can also be integrated with other types of genomic information, such as epigenetic profiles, to gain a more comprehensive understanding of fibroblast heterogeneity. By combining scRNA-seq with techniques like single-cell chromatin accessibility profiling or DNA methylation analysis, the epigenetic landscape of different fibroblast subtypes can be unraveled, providing insights into the regulatory mechanisms that underlie their distinct gene expression profiles and functional roles [39]. DNA methylation, histone modifications, non-coding RNA expression, and chromatin remodeling can be influenced by environmental factors and signaling pathways, ultimately impacting fibroblast phenotype [7,9,10,40]. It has been shown that in mammals, maintenance of a standard DNA methylation status is crucial for normal growth and development of an organism [41] whereas development impairment and disease processes have been associated with abnormal DNA methylation [9,40,41,42]. Senescence in human fibroblasts was shown to be associated with a decrease in the DNA methyltransferase (DNMT) enzymes while immortalized fibroblasts were observed to maintain DNMT [35]. Possible unique and specific markers in chronic impaired wound healing are currently being investigated [9,43]. A recent single-cell RNA sequencing study for instance, was conducted by He et al. [17], providing additional insights into fibroblasts differential gene expression in the process of diabetic wound healing. They highlighted the distinct gene expression patterns among different clusters of CD34+ fibroblasts. These clusters contribute to variations in diabetic wound healing, distinct from those observed in the anagen wound. The anagen wound is characterized by active cellular proliferation, tissue remodeling, and molecular events during the anagen phase of the hair growth cycle. This study led to the identification of 10 different clusters and 6 cell types of CD34+ cells involved in wound healing process including endothelial cells, keratinocytes, immune cells, and 5 subclusters of fibroblasts. Whereas anagen wounds displayed increased proportions of CD34+ fibroblasts C2, associated with up-regulated immune function gene expression, diabetic wounds (DWs) showed elevated proportions of CD34+ fibroblasts C4 with a decrease in fibroblasts C1, which was associated with down-regulated gene expression related to immune pathways.

Most of these characterization studies were conducted either in murine or in human cell lines. Questions regarding the translation from the mouse model to human model continue to be raised. Several investigations have shown evidence in different markers expression pattern between mouse and human species. Dermal fibroblasts markers CD26 for instance, was shown to be mainly expressed in the reticular layer of the adult mice unlike human dermis that exhibits a widespread distribution of CD26+ fibroblasts across the dermal layers [44]. Nevertheless, the emergence of scRNA-seq databases has enabled comparisons between organs in healthy and pathologic states, in both mouse and humans [21] and comparisons between the two species. A close examination with comparative analysis by Buecher et al. [45] revealed conservation of transcriptional states between mice and humans in both healthy and diseased states [45].

Table 1 summarizes dermal fibroblasts heterogeneity on the cellular and molecular levels.

### 2.2. High Plasticity and Adaptation

**Figure 2:**
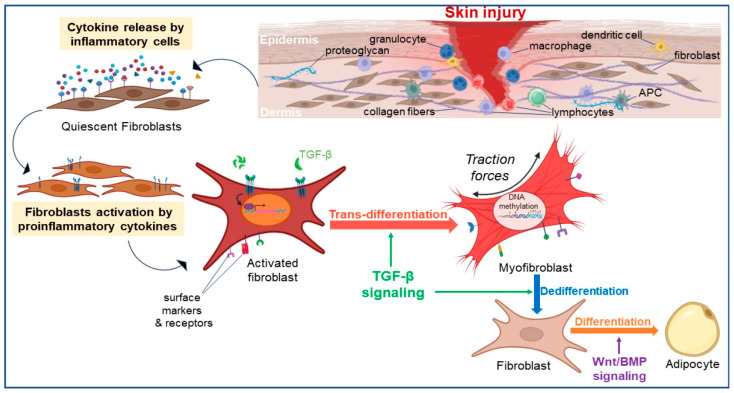
Fibroblasts plasticity during wound healing. Upon injury, fibroblasts exhibit plasticity by trans-differentiation into myofibroblasts that will drive the next phase of the healing process. This activation is mainly regulated by the TGF-β signaling pathway. Epigenetic modifications such as DNA methylation also modulate the mechanism of fibroblasts trans-differentiation. Myofibroblasts’ ability to contract favors closure of the wound. Fibroblasts also can also trans-differentiate into adipocytes during the wound healing process. Myofibroblasts are later cleared from the wound after dedifferentiation into fibroblasts that are later cleared by apoptosis and phagocytosis by macrophages during the wound healing process. TGF-β: Transforming Growth Factor-β.

Fibroblasts demonstrate another key characteristic known as plasticity, indicating their capability to alter their cell phenotype and function in response to diverse stimuli and environmental cues. This plasticity allows fibroblasts to undergo processes like trans-differentiation and dedifferentiation which will be discussed in the following paragraphs (Figure 2).

#### 2.2.1. Fibroblast Trans-Differentiation

Cell trans-differentiation refers to cells regressing to a point where they can switch lineages or phenotype, allowing them to differentiate into another cell type [46]. Under normal physiological conditions, dermal fibroblasts exist in a quiescent or resting state. However, during tissue injury, inflammation, or wound healing, they can undergo phenotypic changes and become activated. In response to mechanical stress and signaling from proinflammatory cytokines and factors upon injury, a cascade of events is triggered during which fibroblasts are activated and undergo phenotypic switch [11,47]. Trans-differentiation into myofibroblasts takes place, resulting in the emergence of a proliferative and contractile cell type that expresses α-SMA. Myofibroblasts display a more migratory phenotype compared to quiescent fibroblasts, and generate mechanical tension, which helps to close the wound by bringing the wound edges closer together. They also produce and deposit extracellular matrix components, such as collagen, to support tissue repair and provide structural integrity. They generate matrix metalloproteinases (MMPs), enable contraction facilitating wound closure, and produce collagen, fibronectin and proteoglycans for the synthesis of new ECM. This structural scaffold serves as a support for the adhesion, migration, proliferation and differentiation of cells, which are essential for the processes of granulation and angiogenesis [6,48,49]. In addition to their contractile and matrix-producing functions, myofibroblasts secrete various regulatory growth factors, cytokines, and chemokines that enable cell communication and recruitment in the wound bed [50]. These include TGF-β, platelet-derived growth factors (PDGFs), FGFs, and connective tissue growth factor (CTGF). These factors stimulate cell proliferation, angiogenesis, and tissue remodeling, promoting the formation of new blood vessels and the recruitment of immune cells to the wound site. Conditions regulating the phenotypic switch of fibroblasts can be intrinsic (cellular) such as cell-cell interactions, or extrinsic (environmental) involving oxygen levels and mechanical forces. Fibroblasts are surrounded by different cell types within the tissue microenvironment, including immune cells, endothelial cells, and epithelial cells, with each of them performing a specific role. Intercellular communication is crucial for the fine-tuning of tissue repair and homeostasis. These interactions can be established either by direct contact of fibroblasts with immune cells and keratinocytes as seen with the Notch ligands for instance, or by autocrine/paracrine manner via differentially expressed signaling molecules such as TGF-β or Wnt signaling molecules derived from immune cells, keratinocytes and endothelial cells [13], as previously described. Oxygen levels within the tissue microenvironment, also known as oxygen tension, can influence fibroblast phenotype. Following tissue injury, hypoxia was shown to stimulate fibroblast activation and the production of factors involved in tissue repair and angiogenesis [51,52]. The behavior of fibroblasts was also demonstrated to be influenced by mechanical forces and physical properties of the tissue microenvironment, with specific ECM components, such as fibronectin, collagen, and integrins playing a modulatory role [6]. Additionally, changes in ECM remodeling enzymes, such as MMPs and tissue inhibitors of metalloproteinases (TIMPs), can regulate fibroblast phenotype.

The process of fibroblast trans-differentiation in the mouse dermis is regulated by a complex interplay of various signaling pathways and molecular factors. 

It is well established that the activation of the TGF-β signaling pathway plays a central role in driving the phenotypic switch of fibroblasts into myofibroblasts [53,54]. TGF-β signaling is a critical pathway involved in both processes of fibroblast activation and dedifferentiation. This signaling pathway stimulates the expression of genes associated with the myofibroblast phenotype, such as α-smooth muscle actin (α-SMA), and fibronectin. This process is accompanied by reorganization of the cytoskeleton and formation of stress fibers, which enable myofibroblasts to exert contractile forces on the surrounding extracellular matrix. Pro-inflammatory mediators like TNF-α, Il-1ß, IL-6 and IL-8, can stimulate fibroblasts to proliferate, produce extracellular matrix components, and acquire specialized functions [1,11]. Several other signaling pathways including PDGFs and members of the Wnt family discussed above, play important roles in promoting fibroblast activation and subsequent dedifferentiation in various physiological and pathological processes. Notch signaling pathway was also shown to be implicated in fibroblast differentiation and wound healing [55]. These signaling pathways interact and crosstalk with each other, forming complex regulatory networks that determine dermal fibroblast behavior and response to the microenvironment. Studies have shown that TGF-β signaling can modulate Notch signaling components, such as the expression of Notch receptors and downstream effectors, influencing fibroblast behavior and fate determination. Conversely, Notch signaling can also regulate TGF-β pathway components, highlighting a reciprocal relationship between these pathways. In DFUs, dysregulated Notch signaling has been reported, thereby affecting fibroblast behavior and function. Shao et al. [49] have characterized the Notch signaling as a molecular switch in determining the plasticity of fibroblasts phenotypic switch into myofibroblasts. This was achieved through an investigation using a genetically modified diabetic mouse model in which the Notch pathway activation is induced exclusively within fibroblasts [49]. This dysregulation is associated with altered expression of key components of the Notch pathway and its downstream target genes (Hes1, Hey1) specifically in the DW environment. The disturbed Notch signaling in DFUs disrupts the normal balance between fibroblast proliferation and differentiation, leading to reduced ECM synthesis and impaired wound healing.

Epigenetic modifications in fibroblasts downstream of these different signaling pathways can also be dynamic and regulate fibroblast plasticity as well, allowing these cells to adapt their gene expression profiles to different stimuli. These modifications can affect gene expression patterns and determine whether fibroblasts remain in a quiescent state or become activated [9,10,11,40].

#### 2.2.2. Fibroblast Dedifferentiation

The process of cell dedifferentiation as defined previously, involves cells becoming less specialized and reverting to an earlier cell state within the same lineage. Fibroblast dedifferentiation often occurs in the process of myofibroblast clearance, a critical step to prevent the persistence of a fibrotic phenotype in which myofibroblasts were found to be apoptosis resistant [56,57]. One of the various mechanisms involved in this step is the activation of MMPs, particularly MMP-2 and MMP-9, that degrade the surrounding matrix, leading to the dismantling and removal of myofibroblasts. Additionally, immune cells, such as macrophages, contribute to myofibroblast clearance by phagocytosing and removing these cells from the wound site [48]. Interestingly, dermal myofibroblasts undergo dedifferentiation in the context of wound healing and tissue regeneration, allowing their clearance. The acquisition of pluripotency provides fibroblasts with heightened plasticity and regenerative properties. This phenomenon has been extensively studied in different organisms, including the axolotl (Ambystoma mexicanum), a salamander with the capacity to regenerate limbs [58,59]. Lin et al. [59,60] reported that fibroblasts in the axolotl’s connective tissue can de-differentiate into a more progenitor-like state, enabling them to contribute to the regenerative process. This dedifferentiation is characterized by the loss of mature fibroblast markers and the reactivation of genes associated with embryonic development and progenitor cell fate [59]. Several factors influence the dedifferentiation and clearance of myofibroblasts involving intricate interplay between different signaling pathways. TGF-β, the potent inducer of myofibroblast activation, is also implicated in initiating the process of dedifferentiation. In an in vitro study on lung fibroblasts, Hecker et al. [60] proposed a model of reciprocal signaling involving TGF-β1/ALK5/MyoD and mitogen(s)/ERK-MAPK/CDKs that regulate the differentiation and dedifferentiation of myofibroblasts. Kosla et al. [61] demonstrated that prolonged expression of certain elements of the MAPK signaling pathway in primary chicken embryo dermal myofibroblasts leads to the inhibition of autocrine TGF-β signaling and the suppression of the myofibroblast phenotype. Furthermore, recent advances in single-cell transcriptomics and lineage tracing techniques have provided insights into the heterogeneity of fibroblast populations in the mouse dermis and their potential for dedifferentiation. These techniques have contributed to Fortier et al. [56] findings that PGE2 induced dedifferentiation of human lung myofibroblasts through the cAMP/PKA signaling pathway, while FGF2 utilized the MEK/ERK pathway, generating transitional cells with unique transcriptomes. Specifically, FGF2 promoted proliferation and survival, whereas PGE2 had an inhibitory effect on these cellular processes [56]. Other signaling molecules, such as PDGF, hepatocyte growth factor (HGF), and interleukin-10 (IL-10), have been implicated in promoting myofibroblast dedifferentiation and facilitating their clearance.

Besides its function in clearance, myofibroblast lineage reprogramming appears to be implicated in the conversion of myofibroblasts to adipocytes in adult mice wounds. Through transcriptomic and functional analysis, Plikus et al. [12,18] were able to provide evidence for the conversion of myofibroblasts to fat cells during the repair process of large wounds, (>1 cm^2^ in size) [12,18]. By employing scRNA-seq approach, this team further identified and characterized a rare subset of highly plastic wound fibroblasts exhibiting myeloid origins, that undergo fat regeneration [18]. This phenomenon was shown to be facilitated by the lineage reprogramming of non-adipogenic wound myofibroblasts through dedifferentiation, regulated by the Bone Morphogenic Protein (BMP) and Wnt pathways [12,18].

Finally, epigenetic modifications downstream of signaling pathways, including changes in DNA methylation and histone modifications, may play an additional role in regulating the dedifferentiation process. Moreover, microRNAs and other regulatory factors are likely involved in modulating the expression of genes associated with myofibroblast activation and clearance [56,57].

In summary, the interactions between various factors involved in fibroblast phenotypic switch can be intricate, and the precise molecular mechanisms governing this process, including myofibroblast reprogramming for either dedifferentiation and clearance, or for differentiation into other cell types, are still being elucidated. Additionally, different tissues and diseases may reveal specific factors contributing to fibroblast plasticity.

### 2.3. Immune Response Modulation

Chronic inflammation is implicated in the pathophysiology of multiple disease processes such as in cardiovascular diseases, cancer, inflammatory diseases, T2DM and chronic ulcers. It thus represents the leading cause of mortality and morbidity worldwide [62,63]. The loss of tissue function that ensues from the persistent inflammation involves impairment of different cell types such as immune cells, epidermal cells, endothelial cells and stromal cells, the latter mostly composed of fibroblasts [23,26,64]. Inflammatory conditions, such as chronic inflammation or tissue injury, can lead to the recruitment and activation of fibroblasts. The immunomodulatory role of fibroblasts is multifaceted. They can attract various immune cells to the site of tissue damage or infection, interact with macrophages, leukocytes, and mast cells. In turn, activated fibroblasts can produce additional inflammatory mediators, including cytokines and chemokines, creating a positive feedback loop. They can interact with immune cells and modulate the immune response in various ways mainly using their inflammatory secretome which includes motifs including (C-X-C motif) ligands (CXCL), C-C motif chemokine ligands (CCL), interleukins, nitric oxide, prostaglandin, TGF-β, cytokines, chemokines, that regulate immune cell behavior, promote tissue healing and regeneration. Dermal fibroblasts possess the ability to interact with immune cells also through expression of toll-like receptors (TLRs) to generate antimicrobials compounds such as defensins. Moreover, the CAF cells described above, contributing to tumor growth and development, were found to have a subpopulation involved in the regulation of T-cell differentiation [23,26]. These immune cells, in turn, can modulate the activity of both keratinocytes and fibroblasts through paracrine signaling, creating a complex interplay between the different cell types. Keratinocytes can produce antimicrobial peptides and release various factors including TGF-β, vascular endothelial growth factor (VEGF)-A and CTGF, that contribute to the innate immune defense of the skin. Fibroblasts can influence immune responses in both pro-inflammatory and anti-inflammatory directions, depending on the context. Once activated, fibroblasts (myofibroblasts) can express surface molecules or produce immunomodulatory molecules like TGF-β and IL-10, which dampen immune responses and promote tissue homeostasis [14,26]. In a healthy organism, this immunomodulatory role is critical to maintain a balanced immune response between immune cells and the surrounding tissue environment by suppressing excessive inflammation and immune-mediated tissue damage. Overall, fibroblasts are versatile cells that originate in large part from mesenchymal progenitors and exhibit lineage-specific characteristics, equipped with remarkable tools to function in variable environments and respond to stress. The complexity of their functions underscores their importance in various physiological and pathological processes in wound healing and tissue integrity restoration. 

## 3. Fibroblasts’ Key Role in Wound Healing

**Figure 3:**
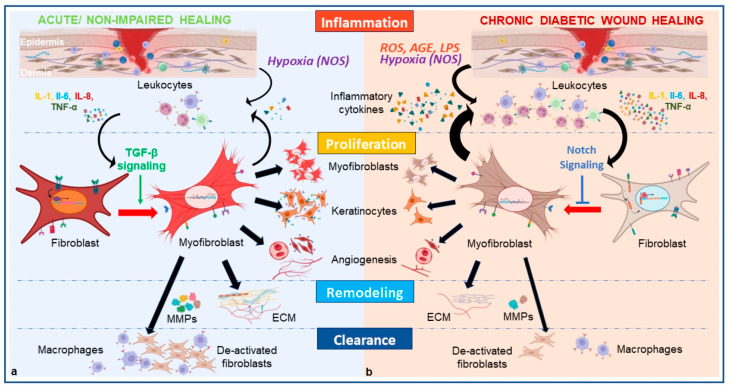
Fibroblasts’ pivotal role in the different phases of acute/non-impaired wound healing vs. chronic diabetic wound healing. Fibroblasts in (**a**) acute/non-impaired wound healing are activated and trans-differentiate into myofibroblasts upon injury responding to hypoxia, after stimulation of the immune system in the inflammation phase by inflammatory cytokines and factors. TGF-β signaling pathway is preferentially activated, regulating the trans-differentiation phase of fibroblasts. Myofibroblasts play a pivotal role in the following phases, secreting additional regulatory growth factors, cytokines and chemokines, stimulating proliferation of additional myofibroblasts, keratinocytes, and endothelial cells, and promoting angiogenesis. Myofibroblasts generate MMPs to break down the old ECM and generate new ECM during the remodeling phase and dedifferentiate into fibroblasts for clearance by macrophage phagocytosis. Fibroblasts in (**b**) chronic diabetic wound healing are impaired by additional ROS, AGEs, and LPSs that overstimulate the immune system, prolonging the inflammatory phase which is further prolonged by preferential activation of Notch signaling that prevents the trans-differentiation of fibroblasts into myofibroblasts. Consecutive phases of proliferation, remodeling, and clearance are consequently altered. AGE: advanced glycation end product; ECM: extracellular matrix; LPSs: lipopolysaccharides. MMPs: matrix metalloproteinases; NOS: nitric oxide synthase; ROS: reactive oxygen species.

Under normal healthy conditions, wound healing is a dynamic process comprising multifactorial, highly orchestrated self-repair mechanisms to restore the integrity of the skin barriers. Four primary overlapping phases occur in regulated succession after acute injury in adults: (1) hemostasis; (2) inflammation; (3) new tissue formation/proliferation; (4) remodeling. Upon injury and loss of the cutaneous barrier, hemostatic mechanisms are activated including the development of fibrin clots and local vascular constriction [65]. Both surrounding tissue at the injury site and fibrin matrix enhance the pro-inflammatory milieu of the wound through the release of mediators through platelet aggregation such as cytokines and FGF, EGF, TGF-β among others [65,66,67]. Upon adequate hemostasis, blood coagulation provides scaffolds for subsequent infiltrating cells. The inflammatory phase proceeds with further secretion of chemotactic signals promoting the upregulation of wound-associate genes from epidermal cells allowing cell migration and infiltration of immune cells into the wound bed. Neutrophils are first recruited to the wound and reach a peak within 24 h, by changing the phenotype and expression of macrophages to generate the innate immune response [68]. They also play a fundamental role in the initial host defense to microbes within the wound bed releasing anti-microbial ROS, peptides, and proteases as well as direct pathogen phagocytosis [1,69,70]. The role of T-lymphocytes in coordinating wound repair is also an intensive area of ongoing investigation. Subsequently, monocytes also appear within wound tissue within 48–96 h after injury and can differentiate into macrophages or polarize into pro-reparative monocyte subsets capable of inducing tissue regenerative mechanisms related to neovascularization, ECM remodeling and controlled fibrosis [19,71,72]. Local and blood-borne fibroblasts proliferate and migrate into the wound area, guided by chemotactic signals, to populate the site and contribute to tissue repair. Macrophages play a particularly important role in the crucial stage of removal of microbial pathogens, apoptotic cells and necrotic cell debris [73]. Evidence has also suggested that they undergo a dynamic process of polarization, which involves the acquisition of distinct phenotypes and functions. Two main phenotypes of macrophages can be found in the wound bed [40]. Pro-inflammatory M1 macrophages are characterized by the production of pro-inflammatory molecule secretion such as TNF-α, Il-1B and Il-6. M1 macrophages also generate ROS and release enzymes that aid in the degradation of pathogens and damaged tissue. They promote inflammation, induce T-cell response to the wound site, and help clear infections. M1 macrophages then polarize to a pro-reparative state M2 macrophages that work towards inflammation regression, processes of angiogenesis, and ECM remodeling [40,73]. The plasticity of macrophages contributes to the transition into the tissue proliferation phase of wound healing by stimulating fibroblasts and keratinocytes under anti-inflammatory factors and cytokine release [13,74,75,76]. They promote fibroblasts phenotypic switch into myofibroblasts [11,47,49], which is a key step to a successful wound healing [48]. Together, these cell types contribute to the immune response necessary to prevent infection and orchestrate the shift into the proliferative phase occurring around 2–10 days post-injury [19]. Myofibroblasts possess contractile proteins, including α-SMA, which allow them to generate mechanical forces. These cells help in wound closure by contracting the edges of the wound, reducing its size. Myofibroblasts play a role in cell proliferation, both through their own replication and by releasing growth factors that stimulate the proliferation of other cells. The interaction between myofibroblasts and these cells including endothelial cells and keratinocytes enables them to form wound granulation tissue, provide structure and signaling cues, and deposit new ECM. Several growth factors, including HGF, one or more members of the FGF and EGF families, modulate the proliferation of fibroblasts and keratinocytes [22,77]. During wound healing, keratinocytes initially migrate to the dermal and mucosal wound surfaces to cover the wound area and form a temporary barrier. They collaborate with fibroblasts to repair the damaged skin and produce various factors including TGF-β, VEGF-A, CTGF, and antioxidants to promote re-epithelialization [38]. Neoangiogenesis, modulated by crosstalk between fibroblasts and endothelial cells [78], and tissue regeneration are mediated by critical interactions between cell populations and the ECM (Figure 3). FGF-1 and FGF-2 produced by various cell types including fibroblasts play a role in angiogenesis as they act on nearby endothelial cells in a paracrine manner. Alternatively, when endothelial cells release FGF-1 and FGF-2, they act on themselves in an autocrine manner, stimulating cell proliferation and differentiation. The administration of FGF-1 has been shown to have a substantial impact on ulcer tissue in diabetes patients [3]. It can effectively increase the quantity of capillaries and fibroblasts present in the tissue. Moreover, FGF-1 enhances the expression of TGF-β [3]. In the creation of the neo-dermis, fibroblasts manufacture collagen as well as component proteoglycans and glycosaminoglycans for ECM synthesis [79]. In the process of granulation tissue formation, FGF-2 facilitates cell migration through surface integrins, which enables the binding of endothelial cells to the ECM. Endothelial cells are also robustly present in forming new capillaries. New capillaries provide necessary blood flow and oxygen to the tissue microenvironment to support fibroblast and cell proliferation [80]. Circulating endothelial progenitor cells (EPCs) can be further recruited to wounded tissue to assist in neovessel formation and tissue regeneration to support fibroblast and cell proliferation [51,81]. The identification of E-selectin, an inducible cell adhesion molecule (CAM) playing a pivotal role in the recruitment and homing of the EPCs and subsequent angiogenesis, provides more insight into this process [82]. Subsequent migration, survival, and differentiation of endothelial cells into capillary morphogenesis is predominantly mediated by signals originating from fibroblasts, as demonstrated by Liu et al. [51]. Following new tissue formation around 2–3 weeks after wounding, the remodeling phase is initiated and can last up to several years [19]. Myofibroblasts become apoptotic [48,56], which results in a dense mass of type III collagen and ECM shaping the wound site. This area subsequently undergoes remodeling by enzymes and MMPs secreted by fibroblasts to decompose collagen, fibronectin and other protein components in the ECM. More type I collagen is then generated to reapproximate the strength of the original cutaneous barrier [19,83].

Given the highly synchronized molecular and cellular programming required to successfully carry out normal cutaneous tissue repair, numerous steps can be inappropriately dysregulated. The two major outcomes resulting from impaired wound healing are either excessive formation of scar tissue (hypertrophic scar or keloid) or an ulcerative skin defect (chronic wound) [11]. In the case of DFU, it appears that tissue damage is caused by a prolonged and sustained inflammatory response resulting from hyperglycemic insult and wound infection [3,22,54] (Figure 3). The fact that fibroblasts found in chronic wounds were characterized by premature senescence, displaying abnormal morphologies, diminished migratory and proliferative abilities as well as impaired capability in trans-differentiation into myofibroblasts [49,84] has warranted further investigation [13].

## 4. Fibroblasts Dysregulation Inherent to the Pathophysiology of DFU

As described previously, wound healing is made possible by the crosstalk between cells of the dermis, the vascular endothelium, the stroma, and the immune system with activated fibroblasts as “maestros” of this phenomenon [13]. Dysregulation of fibroblasts functions and more particularly, dysregulation of their phenotypic switch under hyperglycemic insult in the DW can therefore impact the fine-tuning of the repair process. Dysfunction of fibroblasts during wound healing will yield at multiple levels encompassing the immune response, the progression of angiogenesis, and the deposition of ECM, preventing normal transition from the inflammatory phase to the proliferative phase [1,63,85]. Chronic inflammation is a type of inflammation that persists over an extended period, often lasting for weeks, months, or even years. Unlike acute inflammation, which is a normal and necessary response to injury or infection in which leukocytes are removed from the wound bed tissues during the resolution of the inflammatory phase [54], chronic inflammation is characterized by a prolonged and dysregulated immune response with tissue damage and absence of repair [26]. Prolonged exposure to a high-glucose environment in DFU has been shown to ultimately lead to the development of diabetic complications [64,86,87,88,89]. Nerve damage, hyper viscosity of the blood, accumulation of AGEs, presence of ROS in the ischemic milieu, and higher susceptibility to microbial infections not easily controlled, are all consequences of hyperglycemia contributing to the formation of a persistent ischemic tissue necrosis [29,64,86,87]. Hemoglobin A1c (HbA1c) is a crucial marker used in the diagnosis and management of diabetes, reflecting the average blood glucose levels over the past two to three months. An HbA1c level of 6.5% (48 mmol/mol) or more can be used for a diagnosis of diabetes. There is a significant association between higher HbA1c levels and an increased risk of lower extremity amputation (LEA), such as a 1% increase in HbA1c being associated with a 26% higher risk of LEA [90]. These findings appear to be related to vascular complications.

The mechanisms by which these complications alter the microenvironment of the DFU, are incompletely understood. Evidence from the Diabetes Control and Complications Trial (DCCT) and the observational Epidemiology of Diabetes Interventions and Complications (EDIC) trials, of persistence of gene expression molecular changes after prolonged exposure to glucose reflected by HbA1C levels, even after glycemic control and normalization [40,91], have suggested epigenetic alterations that are now known under the term “metabolic memory” [11,92,93]. Since then, further investigations of metabolic memory have been conducted in different cell types. Park et al. [93] characterized the epigenetic profile of fibroblasts in DFU by performing Genome-wide DNA methylation array signature, in cell lines generated from 3 groups of study participants including, Diabetic Foot Ulcer Fibroblasts (DFUF), Diabetic Foot Fibroblasts (DFF) from diabetic patients without foot ulcers, and Non-diabetic Foot Fibroblasts (NFF) from non-diabetic subjects without foot ulcers [40,93]. Differences in DNA methylation detected after prolonged culture of these diabetic, patient-derived fibroblast cell lines in normoglycemic conditions were observed. They reported decreased level of DNMT1 in DFU fibroblasts associated with a significant reduction of DNA methylation in DFUF in several genes linked to angiogenesis, ECM production and myofibril contraction, which are functions critical to wound healing. This strongly suggests that retained metabolic memory in DFU fibroblasts is associated with poor wound healing outcomes in these diabetic patients [93]. These modifications could notably affect certain genes and pathways that are normally silenced in normal physiological conditions such as the Notch pathway, activated specifically in the DW environment to negatively regulate the fibroblasts phenotypic switch, as characterized by Shao et al. [49] in the mouse model.

The effects of physiological (5.5 mmol/L) and hyperglycemic (25 mmol/L) glucose concentrations on the expression of α-SMA in primary human skin fibroblasts using fibrin gel contraction assay were recently tested by Evangelatov et al. [94]. Their results suggested a lower expression of α-SMA, leading to delayed myofibroblast differentiation and attenuated cell contractility, associated with an inhibited TGF-β signaling pathway, a crucial factor for the regulation of the phenotypic switch in wound repair. The inhibition of the signaling pathway could be the result of several mechanisms resulting from hyperglycemic insult. The identification of distinct gene signatures within CD34+ fibroblast clusters in anagen wounds, as opposed to diabetic wounds [17] is another illustration that highlights alteration of fibroblasts within the hyperglycemic wound milieu.

### 4.1. Hyperglycemia Leads to Phenotypic Switch Alteration via Immune Cell Dysregulation Which Prolongs the Inflammatory Phase

The inflammatory phase relies on an orchestrated sequence of the immune response within the wound bed, with neutrophils arriving first followed by pro-inflammatory M1 macrophages and pro-reparative M2 macrophages after phenotypic shift, and then finally T-lymphocytes [1,54,77]. Growth factors, including PDGF and TGF-β and inflammatory cytokines such as TNF-α are secreted by infiltrating neutrophils and monocytes in the early stage of the inflammatory phase for wound cleansing, and wound contraction is facilitated by myofibroblasts. Low capacity for wound contraction and low generation of granulation tissue have been observed in abnormal wound healing which is associated with an antagonistic effect of TNF-α on TGF-β. Increased levels of TNF-α in DWs have been reported [54] as well as increased levels of other proinflammatory cytokines like (IL)-1β, IL-6, IL-8, in more recent studies [3,54,77,95]. The elevated amount of ROS, AGEs products from necrotic debris as well as presence of lipopolysaccharides (LPS) from biofilm can trigger and maintain abnormally increased levels of local and circulating inflammatory mediators [13,96,97,98]. The secretion of these pro-inflammatory mediators in the DW is thought to be a consequence of the continuous infiltration of monocytes and neutrophils in the wound bed [1,11,13,99] or to be generated by a negative feedback loop from an initial alteration of the innate immune system [100]. This disequilibrium in the DFU milieu appears to be the initial component responsible for a skewed pro-inflammatory M1 macrophage over the pro-reparative M2 macrophages observed in DFU [100,101,102,103]. The interaction between macrophages and fibroblasts is bidirectional and highly influential in regulating the wound healing process. Altered macrophage polarization can have a significant impact on the phenotypic switch of fibroblasts during wound healing which is known to be negatively modulated by TNF-α and positively by TGF-β1. When M1 macrophages dominate the wound environment, they release pro-inflammatory cytokines and chemokines, such as TNF-α, IL-1β, IL-8 and IL-6. The consequent reduction of M2 macrophages in the wound bed lowers the concentration of anti-inflammatory molecules such as Il-4, Il-10 and TGF-β1 which negatively impact the fibroblast phenotypic switch to myofibroblasts [100,103] (Figure 3). Several studies converged towards a particular role of IL-8, known as a chronic wound healing marker, on the plasticity of dermal fibroblasts in DFU [104]. Higher expression of the *CXCL8* gene responsible for IL-8 production, was reported in macrophages and neutrophils within DFU [102]. Littig et al. [105] describe an augmented subpopulation of Il-8 secretory fibroblasts co-expressing CD40/α-SMA contributing to chronic inflammation and impaired healing process, that could counteract the population of myofibroblasts. This reinforces the idea of activated signaling pathways in DFU that could favor proliferation of certain fibroblasts subpopulations while suppressing trans-differentiation of other subtypes into myofibroblasts. Further study of surface markers on the Notch activated fibroblasts in DWs could possibly lead to more sensitive identification methods of a fibroblast population that can be targeted as potential therapeutic targets. Oxidative stress-induced downregulation of neurotrophic factors, including neurotrophin-3 [NT-3], substance P, and calcitonin gene–related peptide (CGRP), described in DFUs [87,89], adversely affects nerve regeneration. These factors also play a crucial role in inflammatory cytokine regulation impacting the typical stimulation of keratinocytes, fibroblasts and endothelial cells observed in normal wound healing [87,89].

### 4.2. Hyperglycemia Leads to Phenotypic Switch Alteration via Dermal Cell Dysregulation and Prevents Angiogenesis and ECM Production

Fibroblasts crosstalk with keratinocytes also regulate the process of wound healing at various stages of the process. Regulation of fibroblasts to myofibroblasts conversion by keratinocytes via TGF-β signaling pathway were highlighted using single cell sequencing transcriptome [22,106]. In the context of DFUs, the communication between keratinocytes and other cells in the wound bed was observed to be disrupted [107]. Several molecular mechanisms involving the impact of hyperglycemia on re-epithelialization and angiogenesis were described. One of these mechanisms is by influencing the activity of transcription factors, such as the Forkhead box O1 (FOXO1) [107]. FOXO1 is a crucial regulator of genes involved in cell cycle regulation, apoptosis, and oxidative stress response. Ponugoti et al. [108], demonstrated its role in acute wound repair, where FOXO1 directly interacts with and modulate the activity of its downstream target TGF-β1, which promotes fibroblasts activation via keratinocyte migration and CTGF secretion [108]. It was later described that molecular changes induced by hyperglycemia and AGEs in DWs can lead to sequestration of FOXO1, binding preferentially to proinflammatory factors, therefore interfering with its TGF-β promoter region interaction [107]. As a result, the alteration of keratinocytes migration and growth factors secretion negatively affects the process of wound healing in the diabetic milieu. Furthermore, FOXO1 is involved in the cellular response to oxidative stress, as it regulates the expression of antioxidant enzymes and stress-response genes. Lineage-specific FOXO1 deletion in keratinocytes was shown to increase oxidative damage in vivo and to potentiate ROS levels, enhancing apoptosis and interfering with keratinocyte migration in vitro [108]. Of note, increased release of IL-8 by keratinocytes in high-glucose environments induced oxidative stress via an ERK-signaling pathway was demonstrated by Lan et al. [109] which could contribute furthermore to impaired fibroblast phenotypic switch.

Investigation of the critical role of Notch signaling in regulating the growth arrest and differentiation of keratinocytes has led Rangarajan et al. [110] to demonstrate that activation of the Notch pathway leads to a significant decrease in keratinocyte proliferation, effectively inducing growth arrest in a keratinocyte-specific conditional deletion of the Notch1 gene mouse model. As previously discussed, Notch signaling is also a critical molecular determinant in regulation of fibroblast trans-differentiation into myofibroblasts [49]. Thus, high intracellular Notch1 activity in fibroblasts suppresses their trans-differentiation into myofibroblasts and impacts proliferation of myofibroblasts interacting cells such as keratinocytes, delaying the process of diabetic wound healing.

One of the reasons for reduced growth factor signaling and fibroblast responsiveness consists of peptide fragmentation via MMPs, the activity of which is enhanced in chronic wounds which creates an imbalance with their tissue inhibitors of metalloproteinases (TIMPs). MMP-1, MMP-8, MMP-9, and activated MMP-2 were found to be significantly elevated in DFU compared to normal wounds in non-diabetic patients whereas levels of TIMP-2 were significantly lower in DFU compared to non-diabetic wounds [3]. DFUs may also exhibit decreased expression of TGF-β receptors, such as TGF-β type I and type II receptors on fibroblasts and keratinocytes as reported by Rai et al. [22], which would render the cells unresponsive to its own signaling. The consequent decreased proliferation and differentiation of fibroblasts into myofibroblasts, due to the impaired communication with keratinocytes, can therefore lead to abnormal ECM remodeling associated with excessive accumulation of certain components such as proteoglycan, and insufficient deposition of collagen. Maione et al. [98] describe an atypical response of fibroblasts in the diabetic wound milieu to TGF-β with an ECM production with abnormal fibrin levels rather than a suppressed response from fibroblasts. This abnormal ECM composition can in turn impair the migration and functioning of keratinocytes, and thereby hinder re-epithelialization.

### 4.3. Hyperglycemia Leads to Phenotypic Switch Alteration via Endothelial Cell Dysregulation

Oxygen and nutritional support to the wound bed are essential components to wound repair. However, impaired angiogenesis in DFU contributes to impaired healing of the diabetic wound. The disrupted balance between ECM synthesis and degradation process in DFU due to decreased fibroblast production as described above, can hinder the migration and proliferation of epithelial and endothelial cells, thereby impairing angiogenesis as well. Fibroblasts are responsible for producing various angiogenic factors such as VEGF, FGF, and PDGF and were shown to play a critical role in vessel morphogenesis [51]. In diabetic foot ulcers, the altered interaction between keratinocytes and fibroblasts can result in reduced production of angiogenic factors, such as VEGF and subsequent impairment of angiogenesis. Additionally, investigation from Siekmann et al. [111] revealed a conserved role for the Notch signaling pathway in limiting the cellular angiogenic response, involving a reduction of VGEF receptors in endothelial cells. This was further supported by the work carried out by Shao et al. [49] that shed light on the downregulation of angiogenesis via IL-6, a functional Notch1 target [22,49]. Moreover, numerous previous studies have shown evidence of TGF-β signaling in angiogenesis partly through the regulation of VEGF [112], some of them suggesting a potential for synergistic effects between Notch and BMP pathways [112,113,114]. This impairment of endothelial cells and angiogenesis secondary to fibroblasts dysfunction in DFU, leads to inadequate blood vessel formation, hampering the delivery of nutrients to the wound site and further delaying wound healing.

## 5. Preclinical Approaches to Treating Diabetic Wounds Using Fibroblasts and Fibroblast-Derived Products

The latest technologies utilized to help treat chronic wounds such as advanced dressings, negative pressure therapy, skin substitutes, and growth factors have shown minimal results, in major part due to the short half-life after administration or potential negative side effects [115]. More advanced and safer biomaterials less subject to degradation, allowing sustained delivery of drugs and instructive signals are therefore highly needed for tissue regeneration. We currently have greater insight into the complex cellular mechanisms and signaling pathways regulating the process of wound healing. Animal models including the mouse, rat, rabbit or pig have been instrumental for the search of novel targets and the conduct of pre-clinical studies. Over 100 genes involved in the process of wound healing were uncovered using the mouse model alone. Expression of the Tac1 gene for instance, coding for substance P (SP), a neuropeptide released early during the process of wound healing regulating mast cells degranulation, was investigated by Leal et al. [116] in both non-diabetic and diabetic mice and neuroischemic diabetic rabbits. Their study indicated that local SP treatment enhances the healing process via modulation of inflammation in the wound bed. This later lead to the topical administration of indole-type a mast cell stabilizer, MCS-01 using alginate bandages to regulate the increased skin MC degranulation observed in diabetes-associated impaired wound healing [117]. In studies on the role of the AKT/β catenin pathway in the differentiation of dermal progenitor cells, a CD34-CrePGR mouse model was utilized [76,118]. This model was subsequently employed for lineage tracing of CD34+ stem/progenitor cells which led to the identification of different CD34+ fibroblasts clusters whether identified in a anagen wound or a diabetic wound [17], as we previously described. These investigations lay the foundation for possible approaches to understand and enhance tissue regeneration by targeting specific CD34+ populations in the diabetic wound. Other models such as the Drosophila, the axolotl and the zebrafish [119] have allowed for the study of highly conserved transcriptional signaling pathways that can be extrapolated to mammals. Study of the modulation of Wnt signaling across diverse species has for example, shown its essential role in enhancing wound healing. Deeper understanding of its reparative mechanisms involving fibroblasts and myofibroblasts holds the potential for innovative therapeutic treatment in regenerative medicine [119]. The growing understanding of dermal fibroblast heterogeneity and their central function in the intricate process of wound healing has led to several approaches utilizing fibroblasts or fibroblasts derived structures, opening avenues for pre-clinical and clinical studies. In-vivo studies conducted by Han et al. [120] have shown an expedited wound healing process in T2DM rats by activating the Akt/β-catenin signaling pathway via subcutaneous injection of dermal fibroblast exosomes [117,120]. They described an improved re-epithelization, angiogenesis, increased collagen deposition and cell proliferation with reduced inflammation [120].

The utilization of cell-based therapies in the treatment of chronic wounds where endogenous cell functions are altered such as in the DW milieu, appears as the treatment of choice. Ongoing preclinical and clinical trials have exploited the delivery of fibroblasts alone or in combination with keratinocytes [20] with minimal clinical efficacy though [117,121]. However, continuous research in the field has led to more advanced therapies comprising cryopreserved placental tissue featuring the placental three-dimensional extracellular matrix (ECM), growth factors, and healthy cells including MSCs and fibroblasts [122,123]. Another novel cell delivery system uses a 3D scanning and 3D bioprinting approach capable of printing healthy autologous fibroblasts and keratinocytes on a collagen and crosslinked fibrinogen scaffold (hydrogel carrier) [119]. This has shown significant results of revascularization and re-epithelization in a murine full-thickness excisional wound model [117,124]. The use of hydrogel with the laminin-derived peptide A5G81 by Ishihara et al. [117,125] in db/db mice with a splint has demonstrated accelerated wound healing by facilitating migration of keratinocytes and dermal fibroblasts, allowing granulation tissue formation and secretion of a new extracellular matrix facilitated by growth factor delivery [125]. Some of these studies have progressed towards clinical trials. An ongoing double-blind study (NCT03880058) is examining the effects fibromudulin (FMOD), a collagen-binding keratan sulfate protein on wound strength and scar appearance. Fibromodulin (FMOD) is a collagen-binding keratan sulfate, which expression is found to be increased in wound healing processes [126]. Preclinical studies in pig models have shown improved dermal collagen architecture organization, reduced scar size and increased tensile strength [117,127].

## 6. Conclusions

The heterogeneity and high cellular plasticity of fibroblasts make them versatile and capable of adapting to various microenvironmental cues. In normal wound healing, fibroblasts transition between quiescent and activated states, allowing them to actively participate in tissue repair processes in a certain time frame. Their ability to secrete growth factors, cytokines, generate ECM components and induce wound closure once activated, makes them crucial for orchestrating the complex interplay between different cell types involved in wound healing. However, persistent hyperglycemia and metabolic memory from the diabetic patient affect fibroblast behavior and their responses to environmental stimuli, leading to dysregulated phenotypic switch. This dysregulation has profound consequences on immunomodulation, angiogenesis, and ECM remodeling, which are essential components of proper wound healing. Understanding the molecular mechanisms underlying the dysregulation of fibroblast plasticity in DFUs and identifying key molecular players as potential targets could pave the way for the development of targeted therapeutic approaches to establish a chronic-to-acute healing switch of DFUs. Nevertheless, the identification of specific subpopulations of fibroblasts within similar tissues undergoing different conditions poses a challenge for the development of targeted therapies. This challenge is further compounded by the presence of comorbidities in diabetic patients, necessitating a more individualized approach to treatment. Addressing chronic DW requires a combined therapeutic approach targeting multiple pathways involved in immune response, angiogenesis, and extracellular matrix remodeling, as these pathways are intricately interconnected. The phenotypic switch of fibroblasts emerges as a critical point of convergence between these various systems. Notably, the pleiotropic effects of the Notch and TGF-β signaling pathways not only regulate fibroblast plasticity but also modulate immune cell responses, keratinocyte differentiation, as well as endothelial cell behavior during angiogenic sprouting. By focusing on modulating these pathways and their downstream effectors while concurrently identifying specific fibroblast markers, it may be possible to unravel the intricate mechanisms governing chronic wound healing in diabetic patients. Understanding the molecular mechanisms underlying impairment of fibroblasts/myofibroblasts in DFU holds promise for the development of targeted therapies tailored to meet the individual needs of patients.

## Figures and Tables

**Table 1:** Dermal fibroblast heterogeneity on the cellular and molecular levels.

Characteristics	Fibroblast Subpopulations	
Morphology	Papillary	Reticular
	Spindle-Shape	Stellate-Shape
Dermal distribution	SDL	DDL
	CD26+	CD26-
	DLK1-	DLK1+
Surface markers	FSP1+	FSP1+
	LRIG1+	LRIG 1-
	PDGFR-α+	PDGFR-α+
Genetic signature	fgf-7	Col1A1
	Itga8	Fbn1

Two main subpopulations of mouse dermal fibroblasts have been identified, based on their morphology, localization in the dermis, cell surface markers, genomic profiles which determine their function. The papillary fibroblast subpopulation is found in the superficial dermal layer (SDL) whereas the reticular fibroblast subpopulation is found in the deep dermal layer (DDL). Several markers were also identified in the human papillary and reticular fibroblasts. Col1A1: Collagen type I alpha 1; DLK1: Delta-like non-canonical Notch ligand 1; Fbn1: Fibrillin 1; fgf-7: fibroblast growth factor-7; Growth Factor-7; FSP1: Fibroblast-specific protein 1; Itga8: alpha-8 integrin; LRIG1: Leucine-rich repeats and immunoglobulin-like domains 1; PDGFR-α: platelet-derived growth factor receptor-α.

## Data Availability

Not applicable.

## Abbreviations

α-SMA	α-smooth muscle actin
AGE	Advanced Glycation End
ALK	Activin-like kinase
APC	Adematous Polyposis Coli
APCs	Antigen Presenting Cells
BMP	Bone Morphogenic Protein
CAFs	Cancer-Associated Fibroblasts
Cav1	Caveolin 1
CDK	Cyclin-dependent kinase
CSL	C-promoter binding factor 1 (CBF1), suppressor of hairless (Su(H)), lin-12 and glp1 (Lag1)
COL1A1	Collagen type I alpha 1
CTGF	Connective Tissue Growth Factor
DCCT	Diabetes Control and Complications Trial
DDL	Deep dermal layer
DFF	Diabetic Foot Fibroblasts
DFU	Diabetic Foot Ulcer
DFUF	Diabetic Foot Ulcer Fibroblasts
Dlk1	Delta-like non-canonical Notch ligand 1
DM	Diabetes Mellitus
DNMT	DNA methyltransferase
DW	Diabetic Wound
ECM	Extracellular Matrix
EDIC	Epidemiology of Diabetes Interventions and Complications
EGF	Epidermal Growth Factor
ERK	Extracellular signal-regulated kinase
FAP	Fibroblast Activation Protein
Fbn1	Fibrillin
FGF	Fibroblast Growth Factor
FSP1	Fibroblast-specific protein 1
FOXO1	Forkhead box protein O1
GSK	Glycogen synthase kinase
HGF	Hepatocyte Growth Factor
IFNγ	Interferon γ
IL	Interleukin
Itga8	Alpha-8 integrin
ITG-β1	Integrin-β1
LEA	Lower extremity amputation
LPS	Lipopolysaccharides
LRIG1	Leucine-rich repeats and immunoglobulin-like domains 1
MAPK	Mitogen-activated protein kinase
MEK	Mitogen-activated extracellular signal-regulated kinase
MMPs	Matrix Metalloproteinases
MyoD	Myogenic Differentiation
NET	Neutrophil Extracellular Trap
NFF	Non-diabetic Foot Fibroblasts
NG2	Neural/Glial antigen 2
NLRP3	Nod-Like Receptor Protein 3
NOS	Nitric oxide synthase
PDGF	Platelet-Derived Growth Factor
PDGFR	Platelet Derived Growth Factor Receptor
Pdpn	Podoplanin
PGE2	Prostaglandin E2
ROS	Reactive Oxygen Species
SDL	Deep dermal layer
T2DM	Type 2 Diabetes Mellitus
TCF/LEF	T-cell factor/lymphoid enhancer factor
TGF-β	Transforming Growth Factor-β
TIMPs	Tissue Inhibitors of Metalloproteinases
TLR	Toll-Like Receptor
TNF-α	Tumor Necrosis Factor- α
VEGF	Vascular Endothelial Growth Factor
Wnt	Wingless-related integration site
